# Creation of New Antimicrobial Peptides 3: Research Promises and Shortcomings

**DOI:** 10.3390/ijms262411992

**Published:** 2025-12-12

**Authors:** Oxana V. Galzitskaya

**Affiliations:** 1 Gamaleya Research Centre of Epidemiology and Microbiology, 123098 Moscow, Russia; ogalzit@vega.protres.ru; 2 Institute of Theoretical and Experimental Biophysics, Russian Academy of Sciences, 142290 Pushchino, Russia

The studies conducted and published in this issue highlight the potential of new peptides as a basis for developing new antibacterial drugs, especially in the face of growing antibiotic resistance [1,2,3]. Recently, they have increasingly been discovered in parasitic organisms or from animal venoms. Thus, a new antimicrobial peptide (AMP) called mesco-2 was discovered in the parasitic flatworm *Mesocestoides corti* [4]. Also, another article is devoted to the design, synthesis and characterization of new chimeric analogs of mastoparan (MP), an antimicrobial cationic peptide from the venom of the wasp *Paravespula lewisii* [5].

Mesco-2 was identified through genomic analysis among three potential peptides (mesco-1, -2, -3). This peptide was found to be highly cationic and amphipathic, which is characteristic of many AMPs [6]. The peptide demonstrated strong activity against Gram-negative (*Escherichia coli*, *Klebsiella pneumoniae*, *Acinetobacter baumannii*, *Pseudomonas aeruginosa*) and Gram-positive (*Staphylococcus aureus*) bacteria, with minimum inhibitory concentrations (MICs) ranging from submicromolar to low micromolar values [4]. Mesco-2 acts by disrupting bacterial membranes, as confirmed by fluorescence microscopy, flow cytometry, and atomic force microscopy (AFM), and does not damage eukaryotic membranes, indicating its selectivity. Circular dichroism spectroscopy and molecular modeling revealed that mesco-2 forms an unusual curved α-helical structure when binding to anionic membranes. The central CRGIGRG motif plays a key role in this structure. The peptide exhibited cytotoxicity only at concentrations significantly higher than antimicrobial concentrations, indicating its safety for mammalian cells.

Mastoparan (INLKALAALAKKIL) is effective against bacteria, but its use is limited due to toxicity (hemolytic activity, mast cell degranulation). The authors of the article created the analogs with improved properties: high antimicrobial activity and low toxicity [5]. Several groups of chimeric peptides were synthesized, combining the mastoparan sequence with other biologically active molecules: analogs with an inverted sequence (retroMP) and their chimeras; chimeras with RNA III inhibiting peptide (RIP), which does not kill bacteria on its own, but suppresses toxin synthesis and cell adhesion in *S. aureus*; chimeras with galanin (Gal) and its fragments, including the known cell-penetrating peptides transportan (TP) and transportan 10 (TP10); conjugates with benzimidazole derivatives attached to the side chain of lysine. The authors determined the minimum inhibitory concentration (MIC) against three reference bacterial strains: *S. aureus*, *E. coli* and *P. aeruginosa*.

A new antifungal peptide, Blap-6, isolated from the hemolymph of the Chinese medicinal beetle *Blaps rhynchopetera*, was presented by the authors of the paper [7]. Blap-6 demonstrates high activity against the pathogenic fungus *Cryptococcus neoformans*, which causes dangerous infections, including meningitis, especially in people with weakened immunity. The minimum inhibitory concentration (MIC) for *C. neoformans* was 0.81 μM, which is 6–8 times lower than that of fluconazole. Blap-6 disrupts the integrity of the fungal cell membrane, as confirmed by transmission electron microscopy (TEM). The peptide also inhibits the formation of *C. neoformans* biofilms and disrupts existing ones, which is important for the treatment of chronic infections. Blap-6 demonstrated low hemolytic activity and cytotoxicity in human cell tests, making it a promising candidate for further research. The peptide consists of 17 amino acid residues (KRCRFRTYRWGFPRRRF) and is rich in arginine and tryptophan, which contributes to its amphiphilic and antimicrobial properties. Its unique structure with an αβ motif distinguishes it from other known peptides of the defensin family. Blap-6 offers a potential alternative to traditional antifungal drugs, such as amphotericin B, which are toxic [7].

Peptides that exhibit multiple biological activities deserve special attention. For example, an ultrashort cyclic peptide of six amino acids cWY6 (WKRKRY) was studied, which has two key properties: antibiotic potentiation and wound healing [8]. Gram-negative bacteria have an outer membrane made of lipopolysaccharide (LPS), which serves as a barrier to many drugs. The authors developed a peptide that does not kill bacteria by itself but disrupts this barrier, allowing common antibiotics to penetrate and act. It is cyclic (head-tail), which increases its stability. Its structure is based on the LPS-binding motif of longer “β-boomerang” peptides developed by the authors previously [9]. It was shown that cWY6 acquires a distinct structure upon binding to LPS. Positively charged residues (Arginine, Lysine) interact with the negatively charged phosphate groups of LPS, while aromatic residues (Tryptophan, Tyrosine) are embedded in the lipid portion of the membrane. This disrupts its integrity. cWY6 significantly enhances the activity of antibiotics that are ineffective against Gram-negative bacteria on their own (vancomycin, rifampin, novobiocin, erythromycin). The effect depended on the bacteria–antibiotic combination, achieving a more than 120-fold reduction in the minimum inhibitory concentration (MIC) for novobiocin against *K. pneumoniae* and *S. enterica*. An unexpected discovery was that cWY6 stimulated cell migration and proliferation (fibroblasts and keratinocytes) in in vitro wound healing models, demonstrating efficacy comparable to natural growth factors. The peptide exhibited very low hemolytic activity and cytotoxicity even at high concentrations. The combination of these properties in a single short, low-toxicity molecule makes it extremely promising for the development of new therapeutic agents.

In the next paper the authors selected polymer conjugates for polymyxin B (PMX B), a peptide antibiotic used against Gram-negative bacteria, to reduce its toxicity, extend its duration of action, and increase its efficacy [10]. All conjugates demonstrated reduced cytotoxicity compared to free polymyxin B. The highest antimicrobial activity (MIC = 4 μg/mL) was observed for the poly(2-deoxy-2-methacrylamido-D-glucose)–PMX B conjugate (PMAG–PMX B) with a hydrolyzable linkage (schiff base). Conjugates with a siderophore (deferoxamine) showed a moderate decrease in activity but remain promising for targeted therapy. Antibiotic release was faster in a slightly acidic environment (pH 5.8), which mimics inflammatory conditions. PMAG proved to be the most promising carrier due to its good biocompatibility, controlled release, and preservation of the antimicrobial activity of polymyxin B. This article represents a significant contribution to the development of antibiotic delivery systems to overcome bacterial resistance and reduce the toxicity of therapy.

Another article is devoted to the development and study of new hybrid antimicrobial peptides (AMPs) created on the basis of amyloidogenic regions of the ribosomal protein S1 of *Staphylococcus aureus*, and demonstration of their effectiveness against pathogenic bacteria, including antibiotic-resistant strains [11]. R23F, R23DI, and R23EI are hybrid peptides containing cell-penetrating peptide (CPP) for penetration into bacterial cells (TAT peptide fragment: RKKRRQRRR) and amyloidogenic sequences from domains D1, D3 and D4 of protein S1 of *S. aureus*. Modifications (sarcosine, aminosuccinimide) have been added to the peptides to increase stability. Antimicrobial activity has been demonstrated against both Gram-positive bacteria (*S. aureus*, MRSA (methicillin-resistant *S. aureus*), *Bacillus cereus*) and Gram-negative bacteria (*E. coli*, *P. aeruginosa*). R23F has the lowest minimum inhibitory concentration (MIC) (75–300 μM depending on the strain). The effectiveness of the peptides varied depending on bacterial strain, emphasizing the importance of specificity. It is believed that the peptides penetrate the cell via CPP and coaggregate with bacterial proteins (e.g., the S1 protein itself), disrupting vital processes. This results in a bacteriostatic or bactericidal effect without direct membrane lysis. Hybrid peptides based on the amyloidogenic regions of the *S. aureus* S1 protein exhibit broad-spectrum antimicrobial activity. They are effective against multidrug-resistant strains (e.g., MRSA). Such peptides are promising candidates for the development of new antimicrobial drugs [12,13,14,15].

The last article in this issue investigates the mechanism of antimicrobial peptide (AMP) penetration through the lipid bilayer of a membrane using molecular dynamics (MD) methods [16]. The authors explore whether computer modeling can predict which peptides most readily penetrate the membrane and what factors influence this penetration. A total of 656 steered molecular dynamics simulations were conducted for 15 peptides. A membrane made of POPC (1-palmitoyl-2-oleoyl-phosphatidylcholine) was simulated. The main conclusions that can be drawn from this work are: the membrane reaction force is maximum at the moment of peptide entry into the bilayer; the lowest reaction was observed for peptides with a high instability index (correlation > 0.9) [17]; the addition of a TAT peptide (cell-penetrating peptide) to the amyloidogenic peptide did not worsen, and in some cases improved, permeability; pulling by the center of mass causes greater drag than by an individual atom due to peptide compaction; the pulling speed (0.01–0.1 Å/ps) did not significantly affect the accuracy of the results. The work does not reveal the actual molecular mechanism of spontaneous penetration of antimicrobial peptides.

Conclusions: It is worth noting that the research conducted in the above-mentioned articles highlights the importance of insects as a source of new antimicrobial compounds and the need to develop safe and effective drugs against resistant bacterial and fungal infections [18,19,20,21,22,23,24,25,26,27]. It is important to consider the synergistic effects of peptides, as demonstrated in recent studies [28,29,30,31,32,33,34,35] and as it works in nature, for example, in frogs, where a cocktail of AMPs appears in their mucus [36]. Particular attention should be paid to peptide stability through the introduction of non-standard amino acids, their modification, or cyclization of the peptide itself [37,38,39,40,41,42,43,44,45,46]. Control peptides are needed that would be composed of the same amino acid residues as the AMPs, but in a randomized order. As emphasized at the very beginning of this summary, peptide and antibiotic resistance is an important and key issue in this area of research [3,47,48].

For practically all peptides, there are no in vivo studies (in animal models) that could confirm their safety and efficacy in the body. Whether these peptides induce resistance in bacteria and fungi during long-term exposure has not been studied. Pharmacokinetics (absorption, distribution, metabolism, and elimination) have not been studied. A more in-depth analysis of the mechanism of action (effect on membrane proteins, risk of resistance) is needed. Comparisons with other peptides should be made to assess competitiveness, not just a narrow range of strains. Before these peptides are considered promising, significantly more in-depth preclinical studies are needed.
